# Supplementary material for: First Report of Candida auris Candidemia in Portugal: Genomic Characterisation and Antifungal Resistance-Associated Genes Analysis
Source: J Fungi (Basel). 2025 Oct 3;11(10):716. doi: 10.3390/jof11100716 (PMC12565043; doi:10.3390/jof11100716)
Supplement: Supplementary file 1 [file jof-11-00716-s001.zip › Supplementary file Table S1.pdf]

**Table S1**. List of *Candida auris* strains used in this study.

| Strain | Clade | Isolation source | Host disease | Country | Assembly Accession | Assembly Name |
|---|---|---|---|---|---|---|
| B13916 | I | blood | Candidemia | United Arab Emirates | GCA_016772235.1 | ASM1677223v1 |
| B11205 | I | wound | - | India | GCA_016772135.1 | ASM1677213v1 |
| B8441 [R] | I | blood | - | Pakistan | GCA_002759435.3 | Cand_auris_B8441_V3 |
| CA-AM1 | I | blood | Bloodstream infection | Italy | GCA_014673535.1 | ASM1467353v1 |
| CA29LBN | I | blood | Candidemia | Lebanon | GCA_019039235.1 | ASM1903923v1 |
| CA21LBN | I | bronchoalveolar lavage | Respiratory infection | Lebanon | GCA_019039275.1 | ASM1903927v1 |
| CA20LBN | I | direct transfer airborne | Respiratory infection | Lebanon | GCA_019039295.1 | ASM1903929v1 |
| CA28LBN | I | blood | Candidemia | Lebanon | GCA_019039315.1 | ASM1903931v1 |
| CA27LBN | I | urine | Urinary tract infection | Lebanon | GCA_019039335.1 | ASM1903933v1 |
| CA26LBN | I | direct transfer airborne | Respiratory infection | Lebanon | GCA_019039355.1 | ASM1903935v1 |
| CA19LBN | I | urine | Urinary tract infection | Lebanon | GCA_019039375.1 | ASM1903937v1 |
| CA15LBN | I | direct transfer airborne | Respiratory infection | Lebanon | GCA_019039395.1 | ASM1903939v1 |
| B11103 | I | urine | - | Pakistan | GCA_031359945.2 | ASM3135994v2 |
| BJCA001 | I | cell culture | - | China | GCA_018831645.1 | ASM1883164v1 |
| L1537/2020 | I | venous catheter tip | COVID19 | Brazil | GCA_020809265.1 | ASM2080926v1 |
| B11220 | II | auditory canal | - | Japan | GCA_003013715.2 | ASM301371v2 |
| B13463 | II | ear fluid | *Candida* infection or colonization | Canada | GCA_016495665.1 | ASM1649566v1 |
| B12043 | II | ear | *Candida* infection | USA | GCA_016495645.1 | ASM1649564v1 |
| B11809 | II | auditory canal | *Candida* infection or colonization | South Korea | GCA_016495685.1 | ASM1649568v1 |
| JCM 15448 | II | - | Otitis media | Japan | GCA_007168705.1 | ASM716870v1 |

| | | | | | | |
|---|---|---|---|---|---|---|
| B12631 | III | arm | *Candida* infection | USA | GCA_016772195.1 | ASM1677219v1 |
| B17721 | III | - | Candidiasis | USA | GCA_016772175.1 | ASM1677217v1 |
| B12037 | III | ear fluid | *Candida* infection or colonization | Canada | GCA_016772215.1 | ASM1677221v1 |
| B11221 | III | blood | - | South Africa | GCA_002775015.1 | ASM3135756v2 |
| A1 | III | cell culture | - | China | GCA_014217455.1 | ASM1421745v1 |
| LOM | III | urine | Urinary tract infection | USA | GCA_005234155.1 | ASM523415v1 |
| BJCA002 | III | cell culture | - | China | GCA_018902005.1 | ASM1890200v1 |
| B11245 | IV | blood | - | Venezuela | GCA_008275145.1 | ASM827514v1 |
| B12342 | IV | stethoscope | - | Colombia | GCA_016772155.1 | ASM1677215v1 |
| B11243 | IV | blood | - | Venezuela | GCA_003014415.1 | Cand_auris_B11243 |
| B11244 | IV | blood | - | Venezuela | GCA_031357835.2 | ASM3135783v2 |
| IFRC2087 | V | ear discharge | Otomycosis | Iran | GCA_016809505.1 | ASM1680950v1 |
| F3485 | VI | nasal, axillae and groin swab | Colonization | Singapore | GCA_032715285.1 | ASM3271528v1 |
| F1580 | VI | back tissue | Infection | Singapore | GCA_032714025.1 | ASM3271402v1 |
| F0083 | VI | blood | Fungemia | Singapore | GCA_032367535.1 | ASM3236753v1 |

The reference strain of *C. auris* is highlighted with a superscript R.
