# Supplementary material for: First Report of Candida auris Candidemia in Portugal: Genomic Characterisation and Antifungal Resistance-Associated Genes Analysis
Source: J Fungi (Basel). 2025 Oct 3;11(10):716. doi: 10.3390/jof11100716 (PMC12565043; doi:10.3390/jof11100716)
Supplement: Supplementary file 1 [file jof-11-00716-s001.zip › Supplementary file Table S2.pdf]

Table S2. Genetic variants detected in *Candida auris* isolates.

Clinical isolate	Single Nucleotide Variants	Deletions	Insertions	Replacements	Multi-Nucleotide Variants
SCO 240	61,058	2,293	3,307	322	3,163
SCO 242	61,345	2,365	3,495	330	3,182
SCO 248	61,617	2,404	3,634	342	3,206
SCO 266	61,437	2,348	3,514	332	3,198
SCO 267	60,619	2,299	3,374	321	3,130
SCO 275	61,659	2,418	3,674	337	3,224
SCO 276	60,666	2,214	3,101	308	3,131
SCO 279	61,058	2,293	3,307	322	3,163
