# Supplementary material for: First Report of Candida auris Candidemia in Portugal: Genomic Characterisation and Antifungal Resistance-Associated Genes Analysis
Source: J Fungi (Basel). 2025 Oct 3;11(10):716. doi: 10.3390/jof11100716 (PMC12565043; doi:10.3390/jof11100716)
Supplement: Supplementary file 1 [file jof-11-00716-s001.zip › Supplementary table S3.pdf]

Table S3. Antifungal minimal inhibitory concentration (MIC) and susceptibility phenotype of *C. auris* isolates according to CLSI guidelines for antifungal susceptibility testing. Strains were classified according CLSI defined ECVs and CDC.

	FLC		VRC		PSC		ISC		ANF		MCF		CSF		AmB	
**Strain**	MIC (µg/ml)	Phenotype ECV/ TBP	MIC (µg/ml)	Phenotype ECV/ TBP	MIC (µg/ml)	Phenotype ECV/ TBP	MIC (µg/ml)	Phenotype ECV/ TBP	MIC (µg/ml)	Phenotype ECV/ TBP	MIC (µg/ml)	Phenotype ECV/ TBP	MIC (µg/ml)	Phenotype ECV/ TBP	MIC (µg/ml)	Phenotype ECV/ TBP
**SCO 240**	>64	ND/ **R**	0.25	ND	0.05	ND	0.03	ND	0.125	WT/ S	0.25	WT/ S	0.5	**NWT**/ S	2	ND/ R
**SCO 242**	>64	ND/ **R**	0.125	ND	1	ND	2	ND	0.125	WT/ S	0.25	WT/ S	0.5	**NWT**/ S	2	ND/ R
**SCO 248**	>64	ND/ **R**	0.25	ND	0.03	ND	0.5	ND	0.5	WT/ S	0.5	**NWT**/ S	1	**NWT**/ S	2	ND/ R
**SCO 266**	>64	ND/ **R**	0.125	ND	1	ND	0.03	ND	0.125	WT/ S	0.25	WT/ S	0.5	**NWT**/ S	2	ND/ R
**SCO 267**	>64	ND/ **R**	0.125	ND	0.25	ND	0.015	ND	0.125	WT/ S	0.125	WT/ S	0.5	**NWT**/ S	2	ND/ R
**SCO 275**	>64	ND/ **R**	0.25	ND	0.03	ND	0.5	ND	0.5	WT/ S	0.125	WT/ S	2	**NWT/ R**	2	ND/ R
**SCO 276**	>64	ND/ **R**	0.125	ND	0.125	ND	0.015	ND	0.125	WT/ S	0.125	WT/ S	0.5	**NWT**/ S	2	ND/ R
**SCO 279**	>64	ND/ **R**	0.125	ND	0.03	ND	0.5	ND	0.25	WT/ S	0.25	WT/ S	1	**NWT**/ S	2	ND/ R

FLC- Fluconazole; VRC – Voriconzole; PSC – Posaconazole; ISC – Isavuconazole; ANF – Anidulafungin; MCF – Micafungin; CSF – Caspofungin; AmB – Amphotericin B
Phenotype (Phen) at 24 hours incubation, based on CLSI epidemiological values (ECV) and CDC tentative breakpoints (TBP). ND - not defined; S – Susceptible; R – Resistant;
